# Shoreline Detection and Land Segmentation for Autonomous Surface Vehicle Navigation with the Use of an Optical System

**DOI:** 10.3390/s20102799

**Published:** 2020-05-14

**Authors:** Stanisław Hożyń, Jacek Zalewski

**Affiliations:** Faculty of Mechanical and Electrical Engineering, Polish Naval Academy, 81-127 Gdynia, Poland; j.zalewski@amw.gdynia.pl

**Keywords:** shoreline detection, land segmentation, marine navigation, autonomous surface vehicle

## Abstract

Autonomous surface vehicles (ASVs) are a critical part of recent progressive marine technologies. Their development demands the capability of optical systems to understand and interpret the surrounding landscape. This capability plays an important role in the navigation of coastal areas a safe distance from land, which demands sophisticated image segmentation algorithms. For this purpose, some solutions, based on traditional image processing and neural networks, have been introduced. However, the solution of traditional image processing methods requires a set of parameters before execution, while the solution of a neural network demands a large database of labelled images. Our new solution, which avoids these drawbacks, is based on adaptive filtering and progressive segmentation. The adaptive filtering is deployed to suppress weak edges in the image, which is convenient for shoreline detection. Progressive segmentation is devoted to distinguishing the sky and land areas, using a probabilistic clustering model to improve performance. To verify the effectiveness of the proposed method, a set of images acquired from the vehicle’s operative camera were utilised. The results demonstrate that the proposed method performs with high accuracy regardless of distance from land or weather conditions.

## 1. Introduction

Autonomous surface vehicles (ASVs) have received attention recently due to their application in many fields, including military operations, environmental protection, coastal guard patrol inspection and sea rescue [1]. Thus, numerous research projects have been devoted to developing autonomous vehicles that carry out missions with minimum to no human supervision. According to “Global Mar. Technol. Trends 2030”, published by Lloyd’s Register of Shipping and Southampton University, autonomous intelligent vehicles are listed as one of the eight critical marine technologies of the future [1].

Autonomous surface vehicles require navigation in coastal areas with a safe distance from land using available sensors and devices. For this purpose, a navigation radar, a laser rangefinder and a LIDAR (Light Detection and Ranging) system are utilised [2]. They provide essential data for trajectory determination and obstacle detection, which are useful for safe navigation [3]. However, these data are insufficient for perceiving topographical information. To compensate for this, visual information obtained using optical systems is needed. 

The optical systems need complex image processing algorithms to perform their required sophisticated tasks [4]. Therefore, algorithms devoted to image segmentation, object detection, pattern recognition, as well as feature extraction and classification, have been developed [5]. Most of them are based on traditional image processing methods, which depend on domain knowledge to analyse images. Another approach applies artificial intelligence tools, typically neural networks, to understand and interpret a given scene [6]. 

Optical systems have been deployed in ASVs for horizon line detection [7,8], water line detection [9], sea–sky line detection [10,11], sea–land line detection [12,13], and obstacle detection [14,15]. Their applications have focused on the ship’s orientation determination, trajectory planning and navigation. Another employment of optical system has been presented in [16], where a vehicle position in a coastal area can be determined using vision systems. To this end, the distance from the land, as well as its size, should be estimated. Then, the obtained information can be connected to the electronic chart system to estimate the vehicle’s geographical position [17]. 

Shoreline detection is used to calculate the distance from land. However, previous shoreline detection research has used satellite imagery to monitor costal systems. In line with this, some researchers have resolved the problem of horizon line detection for ASVs. Shoreline detection for optical navigation was only mentioned in [17]. Nevertheless, since plenty of horizon line detection methods are based on recognition of the most distinguishable line in the image, they can be utilised for shoreline detection under the assumption that shoreline is viewed as a straight line in the vehicle’s camera. Due to the fact that this assumption was adopted in this work, horizon line detection methods were taken into consideration. For example, the method based on Otsu segmentation and Hough transform has been previously utilised in [11], where the longest straight line is considered as a horizon line. Gradient techniques, utilising the Canny filter, were introduced in [18,19]. In these methods, the Hough transform is also implemented in the line determination step. In [8], a robust horizon line detection method, named coarse-fine-stitched and based on hybrid features, was proposed. It is divided into three steps: the coarse step, devoted to pointing all lines in the image; the fine step, to select the horizon line’s segments; the stitching step to obtain the whole line. 

A horizon line detection method, based on the K-means technique, was presented in [20]. In this approach, the image is divided into clusters, where the horizon line is determined using the least-square method. Since some nonconnected pixels can remain after clustering, the union-find algorithm is applied. Another approach, utilising a quick algorithm for horizon line detection, was presented in [7]. It employs the iterative approach to define the probable location of a horizon line. Then, a comparison of the brightness of parallel lines lying in a distinguished region of interest (ROI) is analysed. Consequently, the highest difference in brightness is considered as a horizon line. This method was also utilised for shoreline detection as the first step of land segmentation in [17]. A multiscale approach has been previously utilised in [21]. In this study, the MSCM-LiFe technique is based on the Hough transform and the intensity gradient to find the line candidates. The method appears to be efficient for line segment extraction but demonstrates a less adequate performance when similar line segments appear in a sea area. Another approach, utilising a statistical algorithm based on constrained unsupervised segmentation was presented by Kristan et al. [22]. This technique employs a semantic segmentation model (SSM) for structurally constrained semantic segmentation. Global gradient saliency was utilised by Wang et al. [10] to detect a sea-sky-line position. In this technique, global features are used to calculate an optimisation criterion based on the features in the in the totality of the image. However, the presence of the inconsistent surface motion of the sea can influence the accuracy of this method. 

Land segmentation also plays an important role in ASV’s applications. It can be defined as an image segmentation problem, which represents an important branch of image processing. Due to this, numerous algorithms to divide an image into regions have been developed. Most of them use traditional image processing methods, such as the Flood Fill, Watershed, Grabcut, Mean shift and K-means [5]. Another approach implements semantic segmentation based on convolutional neural networks. Traditional methods have evolved for many years, and their efficiency has been acknowledged in many applications. However, their utilisation often relies on a number of parameters, which should be correctly set for the best performance. Semantic segmentation, on the other hand, demands a lot of data to execute the learning process. What is more, its performance strongly relies on a designed network architecture [23].

A land segmentation algorithm, dedicated to ASVs and based on traditional image processing, was proposed in [17]. In this approach, segmentation is performed in two steps: firstly, a straight line separating land and sea is determined, and secondly, a line separating land and sky is distinguished. In the first step, the horizon line detection algorithm is utilised. The second step deploys the gradual edge level decrease algorithm, which is committed to finding a path between the left and right side of an image, representing a land–sky boundary. Even though the method is useful for land segmentation, its performance depends on the set of parameters, which should be tuned before execution. 

A semantic segmentation-based approach was presented in [24]. It employs an encoder–decoder convolutional network, which is initialised by labelled images. Then, the super pixel-based refinement algorithm is deployed to label images for the self-learning process. Finally, the uncertainty evaluation criteria for measuring the confidence of pixel prediction are applied. This method starts with a limited performance due to small training data. However, the performance, which uses images acquired by the ASV gradually improves during the self-training step. Another approach utilises the multistage segmentation algorithm, as described in [25]. In this process, a lidar sensor is used to perceive the obstacle in front of the ASV. To perform it, the spatial relationship between the lidar sensor and the camera has to be determined. Then, the segmentation results are conveyed to a convolutional neural network as training examples. At the final step, the modified binary cross-entropy loss function with the weight map is used to assess reliability during the network training.

Overall, the methods mentioned above for ASV applications employ a set of parameters or labelled images. To overcome these weaknesses, we developed a traditional approach based on progressive segmentation, which utilises only few parameters in order to be executed. The values of these parameters were established during experiments, and they are valid for all pictures in the database. Consequently, there is no need to adjust them during ASV operations. According to the obtained results, this method detects shoreline with 99% accuracy and segments land with 96% accuracy. The results indicate a high level of confidence in this method, making it a suitable one for ASV applications.

The remainder of this paper is organised as follows. The details of the proposed method are presented in Section 2. Section 3 discusses the experiments conducted to evaluate the practical utility of this approach. Finally, the conclusions are included in Section 4.

## 2. Methodology

There are two goals to this method. First, to detect a shoreline, and second, to segment land. To this end, based on traditional computer vision techniques, an image processing algorithm was developed. It can be divided into the following interrelated components:

- Image pre-processing;
- Edge detection;
- Shoreline detection;
- Progressive land segmentation.

Image pre-processing aims to convert an RGB image into a grayscale one. Edge detection, based on a greyscale image, utilises adaptive filtering to distinguish strong edges, while shoreline detection uses obtained edges to determine a shoreline location. Finally, land segmentation based on detected edges is performed. The algorithm is described in detail below. 

### 2.1. Image Pre-Processing

**Figure 1:**
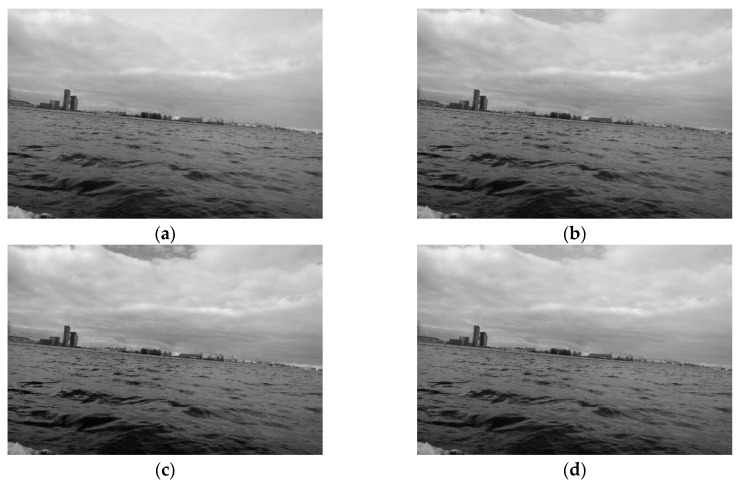
(**a**) Channel V of the HSV colour model; (**b**) Channel B of the RGB colour model; (**c**) Channel R of the RGB colour model; (**d**) A grey channel obtained using the conversion formula.

An image pre-processing step splits the RGB colour space into a greyscale one, which is necessary for the method. For a greyscale space, a conversion formula technique is needed, in which the grey image is obtained as a weighted addition of red, green, and blue channels in ratio 0.21:0.72:0.07. Another solution is based on separating channels from various colour spaces [26]. Therefore, in our work, we tested the individual channels from the RGB, LAB, YCrCb and HSV colour spaces as well as the conversion formula. Some greyscale images are presented in Figure 1.

Our research indicates that the selection of the channel slightly influences the performance of the algorithm. As can be seen in Figure 1, the sky area in the picture (a) is less cloudy than the sky areas in the other images. Consequently, fewer edges are detected in the sky region. Strong edges in the sky area, in particular those which are connected to the land region, can impede land segmentation. Therefore, Channel V of the HSV colour model, which can reduce the number of edges, was chosen as the best choice to be employed for the method.

### 2.2. Edge Detection

Edge detection constitutes a crucial point of the method. In order to facilitate land segmentation, it should reliably detect strong edges and suppress weak ones. Therefore, to assign the most reliable edge detector, there was a consideration for the techniques of Prewitt, Sobel, Canny, and Laplace [27]. The research suggests that the Prewitt, Sobel and Laplace detectors are unsuitable because they did not suppress weak edges. The Canny detector produces the best results, but it needs threshold values, which should be determined for each image separately. In addition to that, the Canny detector did not ensure the lines’ continuity, a much-needed factor for segmentation [28]. To overcome these difficulties, the adaptive filtering technique was introduced.

In this approach, we assume that the analysed window of the image can be expressed as

(1) $$J=\left|\begin{array}{ccc} a_{0} & a_{1} & a_{2}\\ a_{3} & a_{4} & a_{5}\\ a_{6} & a_{7} & a_{8} \end{array}\right|,$$

where *a_i_* is the *i-th* pixel value in the region.

**Figure 2:**
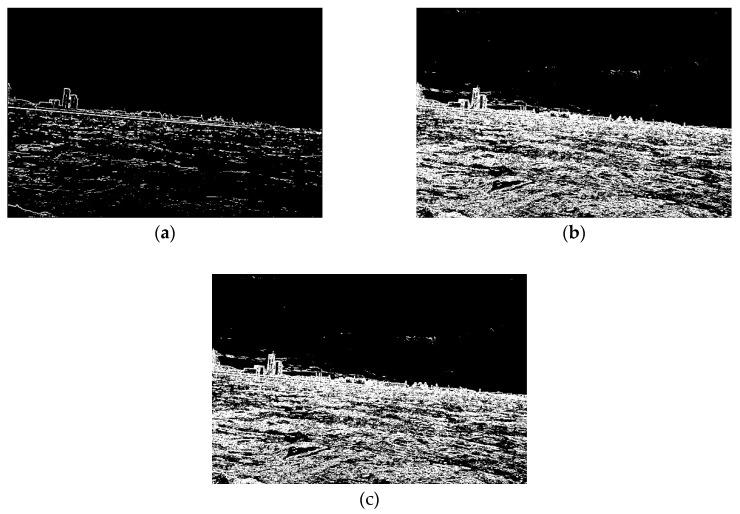
Results of edge detection (**a**) Adaptive filter; (**b**) Sobel operator; (**c**) Prewitt operator.

Defining the input mask of the filter

(2) $$F_{AM}=\left|\begin{array}{ccc} 1 & 1 & 1\\ 1 & 1 & 1\\ 1 & 1 & 1 \end{array}\right|,$$

the filter mask can be represented as

(3) $$H=J-\bar{m}F_{AM},$$

where H is the filter mask, and $\bar{m}$ is the mean value of pixels in the window ($\bar{m}=\frac{1}{9}\sum_{i=0}^{i=8}a_{i}$). The filter mask is calculated for every pixel in the input image. Based on experimentation, it was found that the adaptive filter better suppresses weak edges than gradient filters like Prewitt and Sobel. In Figure 2, the comparison between the adaptive filter as well as Sobel and Prewitt operators are depicted. For the Sobel and Prewitt operators, the following kernels were employed: [1, 2, 1; 0, 0, 0; −1, −2, −1] and [1, 1, 1; 0, 0, 0; −1, −1, −1], respectively. In the case of the adaptive filter, the kernel is calculated for each pixel in the image individually. The suppression of weak edges is connected to the fact that the mask coefficients of the adaptive filter depend on the mean value of neighbouring pixels. Consequently, if edges are not presented in the considered region, the response of the filter is smaller than in the case when edges exist. For the same reason, the adaptive filter is less sensitive to noise than others [27]. To facilitate comparison between the filters, the resulted images were binarised with a threshold equals 10. As can be seen in Figure 2, for the adaptive filter, fewer weak edges are presented compared to the Sobel and Prewitt operators. Therefore, the adaptive filter is implemented in the method under consideration. 

**Figure 3:**
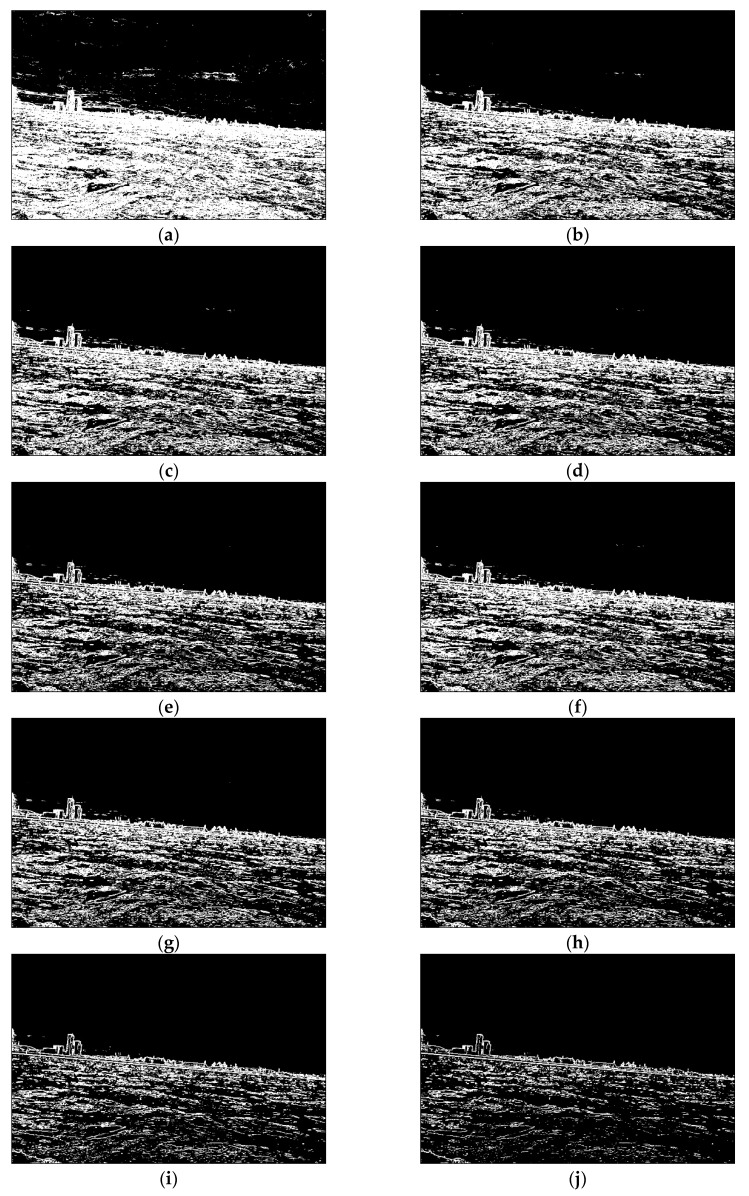
Results of edge quantization (**a**) Pixels equal 0; (**b**) Pixels equal 1; (**c**) Pixels equal 2; (**d**) Pixels equal 3; (**e**) Pixels equal 4; (**f**) Pixels equal 5; (**g**) Pixels equal 6; (**h**) Pixels equal 7; (**i**) Pixels equal 8; (**j**) Pixels equal 9.

With the implementation of the edge detection step, the image pixels are normalised, so their values are set between 0 and 1. In the next step, quantisation is performed; as a result, the pixels are divided into *T* groups, in which each pixel gets a new value from 0 to *T,* proportionately to its input value. In this approach, *T* is a positive integer number ($T=0,1,2,\ldots ,T_{max}$), while $T_{max}$ was determined during experiments to be equal to 15. Higher values did not influence the obtained results. Consequently, the new image is composed of pixels, which are rescaled from the range 0–1 to the values between 0 and *T*. This approach plays a crucial role in shoreline detection and, consequently, land segmentation. In Figure 3, the respective groups of pixels are presented (for *T* equals 10). As can be seen, the figures with lower pixel values contain more edges than figures with higher pixel values. This quality is utilised during shoreline detection and land segmentation, where weak edges are important for land segmentation while strong edges facilitate shoreline detection.

### 2.3. Shoreline Detection

Shoreline detection is based on the images obtained in the edge detection step. We define a line as a shoreline in the case when it separates land and sea areas. Due to the fact that, according to project assumptions, the vehicle will utilise optical navigation at a greater distance from land, we established that the shoreline can be represented as a straight line across. Additionally, we assumed that, in case the shore area is not visible on the image, a line separating land and sky area represents the shoreline. Consequently, a shoreline should have the following characteristics:

- It should be the most distinguishable line in the image,
- It should constitute the most extended line among the most distinguishable ones.

To fulfil the first condition, for each line connecting the left and right sides of the image, its strength is calculated according to

(4) $$L_{St}=\sum_{i=0}^{i=n}b_{i},$$

where *n* is the number of pixels belonging to the line and *b_i_* is the *i-th* pixel value.

In our research, we assumed that subpixel accuracy is not necessary; consequently, each line is determined using its ending positions in the first and last columns. Based on them, the line position in each column is calculated from the formula y=ax+b, where a is a slope, b is an intercept and $x=1,2,3,\ldots ,x_{max}$ where $x_{max}$ is equal to the horizontal resolution of an image. This means that a number of pixels belonging to the line is the same as the horizontal resolution of an image. Consequently, the lines with the largest strength are considered as the most distinguishable ones. To implement the second condition, an additional coefficient *L_Long_* was introduced

(5) $$L_{Long}=\frac{\sum_{i=0}^{i=n}C_{i}}{n},$$

where *n* is the number of pixels belonging to the line and *c_i_* is defined as

(6) $$c_{i}=\{\begin{array}{ll} 0 & if\;b_{i}<0.75T\\ 1 & otherwise \end{array}.$$

**Figure 4:**
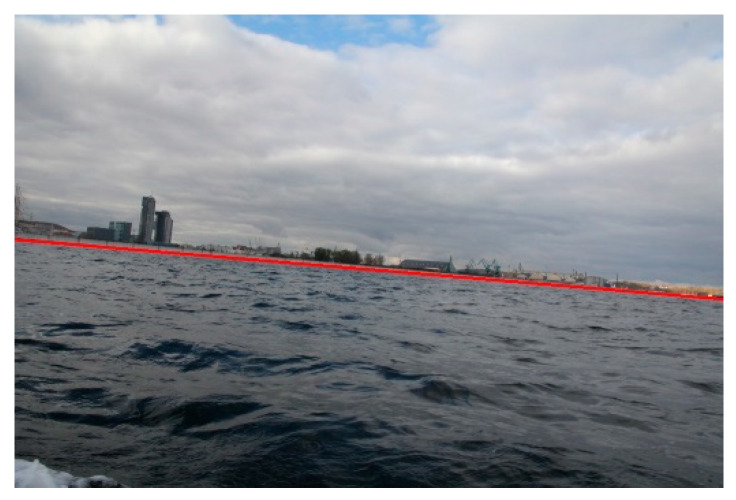
Results of shoreline detection.

Finally, each line is characterised by coefficient *L*

(7) $$L=L_{St}L_{Long},$$

and a line with the highest *L* value is considered as a shoreline. Figure 4 illustrates the results of shoreline detection for the exemplary image. 

**Figure 5:**
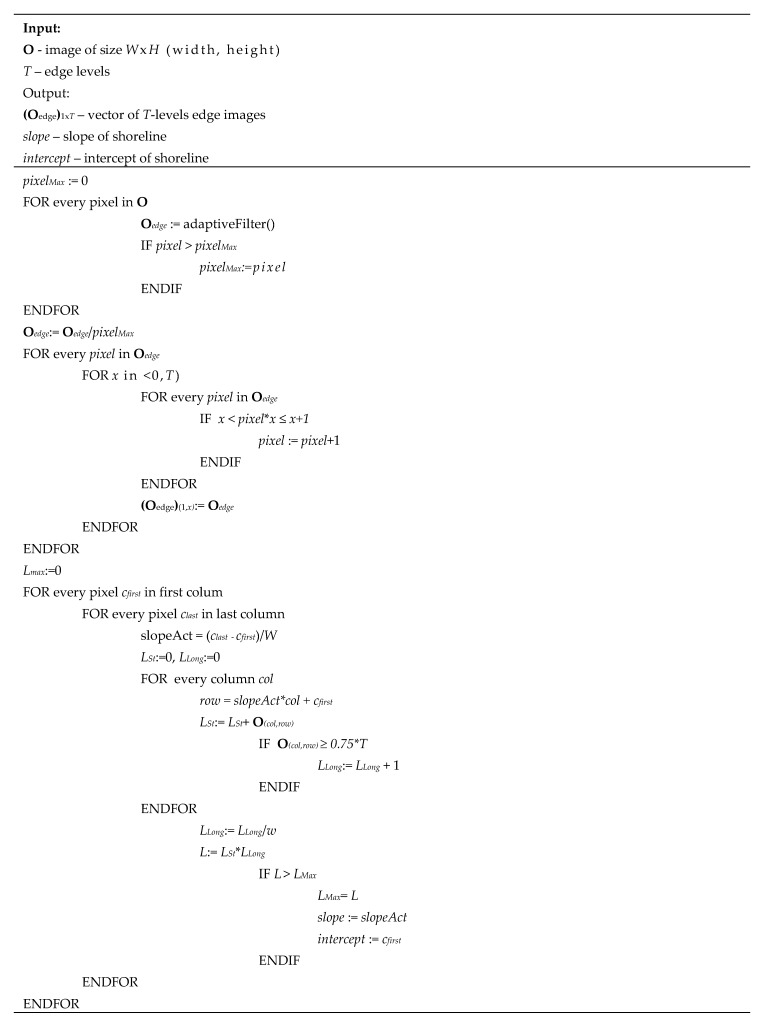
Pseudocode of shoreline detection.

A pseudocode of the proposed algorithm is given in Figure 5.

### 2.4. Progressive Land Segmentation

**Figure 6:**
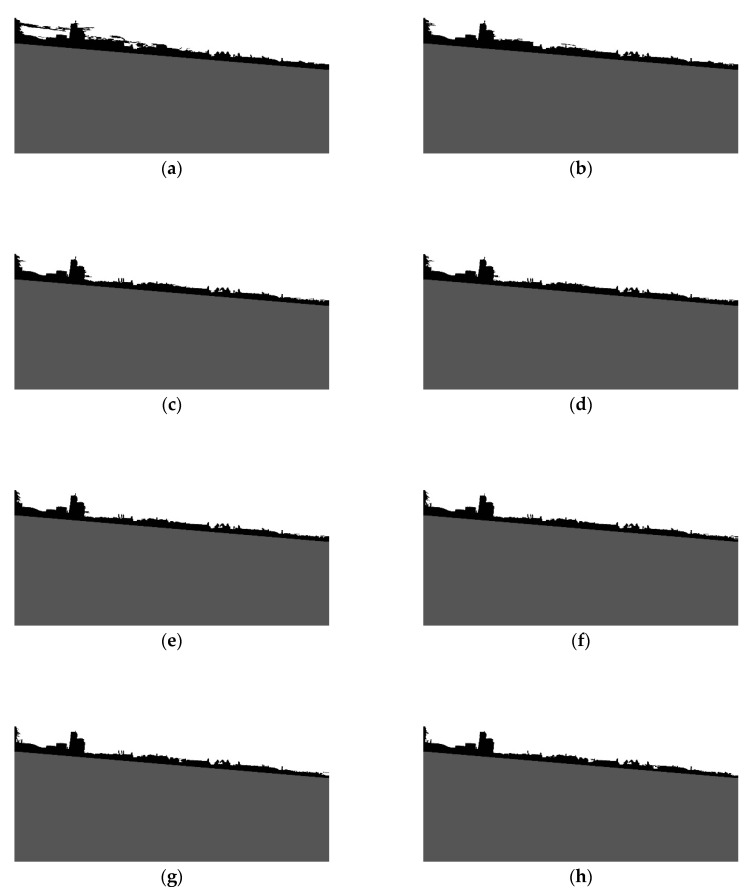
Results of progressive segmentation: (**a**) Level 0; (**b**) Level 1; (**c**) Level 2; (**d**) Level 3; (**e**) Level 4; (**f**) Level 5; (**g**) Level 6; (**h**) Level 7.

Since the shoreline was detected in the previous step, the sea area can be excluded from the image. As a result, only land and sky should be separated. To accomplish this, progressive land segmentation technique was implemented. In this technique, we assume that the land area constitutes the biggest cluster of connected pixels, located next to the detected shoreline. Therefore, in the first step, the land area is determined using the image with pixels values equal to 0, obtained during edge detection (Figure 3a). The pixels are then connected into clusters, and the biggest one is considered as the land area. The remaining clusters are removed from the image. At the segmentation stage, the connected-component algorithm is the most applicable option to use [29]. Since the image contains only one cluster of pixels representing the land area, the pixels above that cluster are grouped together to constitute the sky area, and therefore are marked as such. The whole process is repeated for all pictures obtained in the edge detection step (see Figure 3). Consequently, *T* levels of land segmentation are obtained (levels 0–7, for *T* equals 10, were depicted in Figure 6).

**Figure 7:**
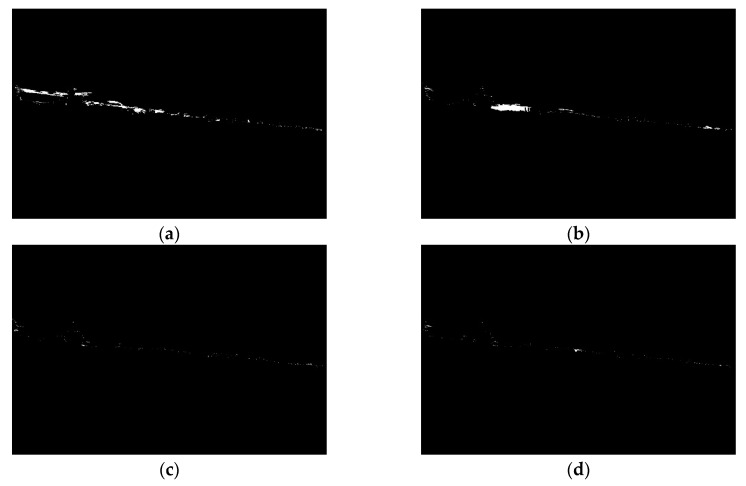
Removed land areas (**a**) at Level 1; (**b**) at Level 2; (**c**) at Level 3; (**d**) at Level 4.

As can be seen, at each level some part of the land is removed. This is because the weak edges at each level gradually disappear, allowing the sky area to expand. In the majority of tested images, the proper land area is obtained in lower levels of progressive segmentation. At higher levels, land and sky become indiscernible, since some edges inside the land area are stronger than the edges separating the sky and land areas. Therefore, based on these previous findings, we decided that 0.5*T* levels of progressive segmentation should be executed. Figure 7 illustrates the removed land area in four steps of progressive segmentation.

**Figure 8:**
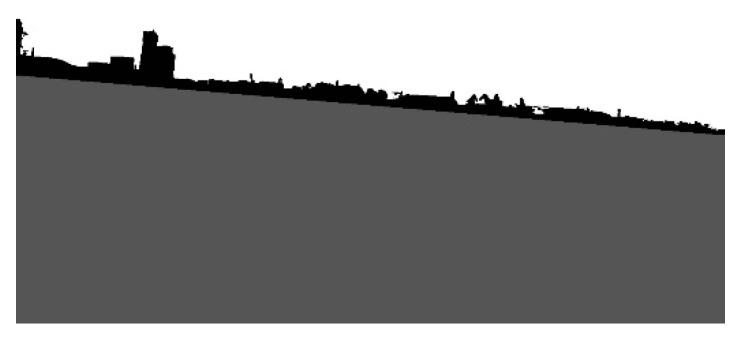
Results of the progressive segmentation.

Although in most of the cases the sky areas were properly removed, in some instances, especially when land was located far away from the camera, the process was executed incorrectly. To remedy this, we have formulated a method utilising a probabilistic clustering model, which is devoted to establishing if a defined area should be excluded from the land area. In this approach, we assume that, for each pixel in the removing area, the probability of belonging to sky or land is estimated. To perform this, the following probability density function is applied

(8) $$p\left(x_{i}|z_{i}=k,\mu_{k},\sigma_{k}\right)=N\left(x_{i},\mu_{k},\sigma_{k}\right),$$

where $x_{i}$ denotes *i-th* pixel in the image, *k* = {*land*,*sky*}, *µ_k_* is the mean of pixel distribution, and *σ_k_* is the standard deviation in land or sky area. The means and standard deviations were calculated for areas defined during progressive segmentation, excluding the examined area. Then, the pixels were jointed into clusters, and if most of the pixels in the cluster have a higher probability of belonging to sky, the cluster was removed from the land area. Figure 8 displays the result of the progressive segmentation.

**Figure 9:**
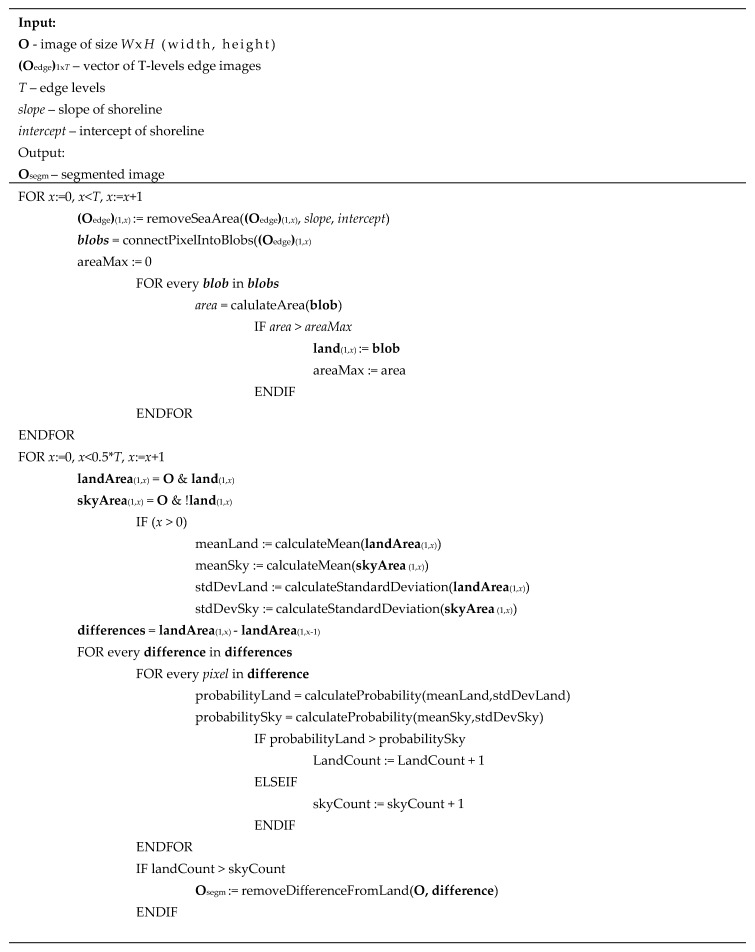
Pseudocode of progressive land segmentation.

A pseudocode of the progressive land segmentation algorithm is presented in Figure 9.

## 3. Results and Discussion

**Figure 10:**
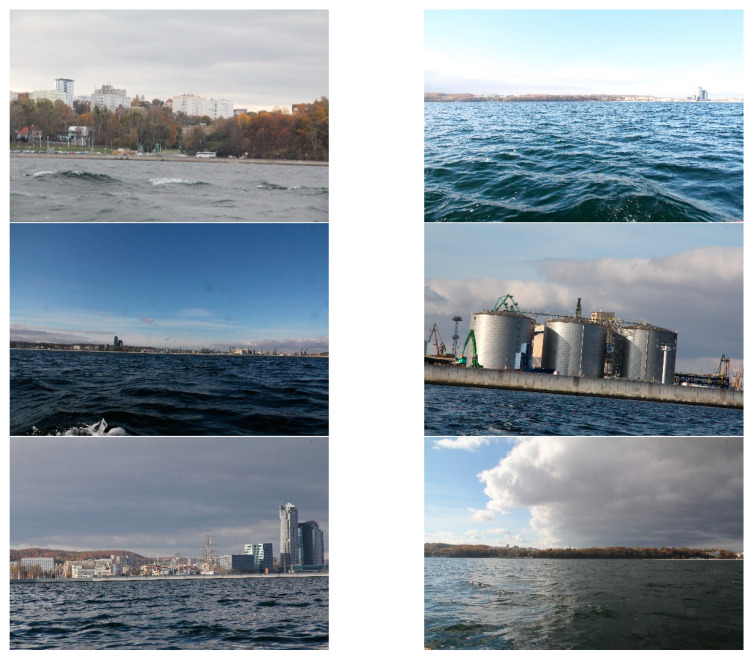
Examples of the images used during the experiment.

The proposed method was developed for shoreline detection and land segmentation for the ASV navigation. Since the method will be implemented in a practical solution, we were interested in testing it under the conditions which we expect in a real environment. After analysing the most popular open source datasets, such as Singapore Maritime Dataset (SMD) and Marine Obstacle Detection Dataset (MODD), we concluded that majority of images included in these databases present open sea views or show lands from short distances. Because our vehicle will operate at more considerable distances from land, utilising a view of the land for navigational clues, we decided to build our database. Consequently, we captured thousands of images from the planned operating site of the vehicle near Gdynia city. The images show coastal areas at different distances from land (up to 3 km) and under various weather conditions. One thousand five hundred (1500) of them were selected and utilised to verify the reliability of the presented method. Six of these images, as a sample, are presented in Figure 10. 

**Figure 11:**
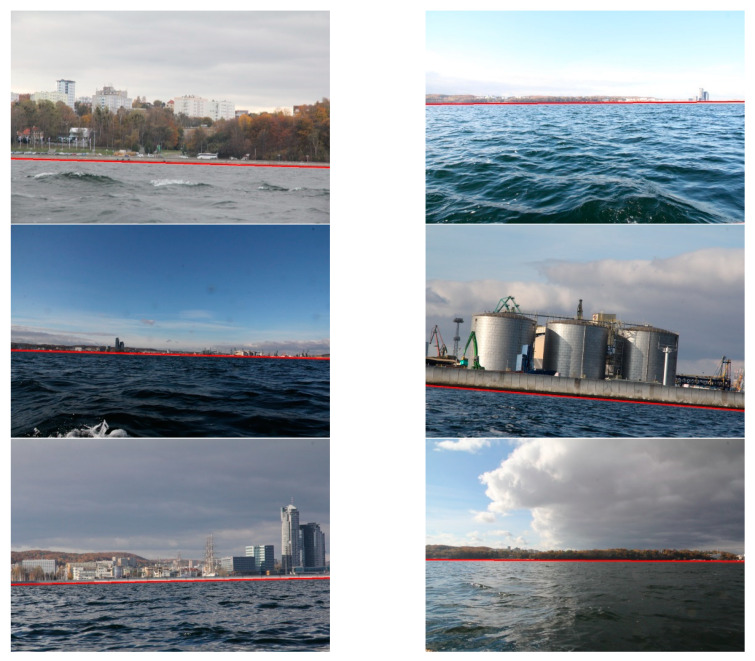
Results of shoreline detection.

Firstly, the shoreline detection was taken into consideration. Since the method needs the *T* parameter to be executed, preliminary experiments were devoted to determining its optimal value using 10% images from the database. Afterwards, the obtained result was tested utilising the whole database. Consequently, we established that *T* equals 10 gives the best results for all images in the database. The images were divided into three groups: close (up to 1 km), medium (2–3 km), and long-distance (above 3 km) from land. For each group, a human eye examined the shoreline detection to verify correctness. The results were qualified as correct when a distinguished line approximately covered the line appointed by a human (maximum difference amounted up to 10 pixels). Some of them are displayed in Figure 11.

The quantitative results, summarised in Table 1, demonstrate that the method distinguishes shorelines correctly.

**Figure 12:**
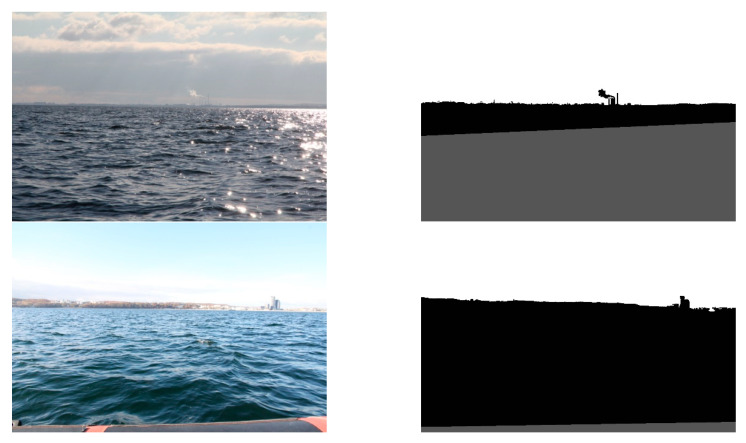
Results of incorrectly detected shorelines.

Two cases of incorrect detection are presented in Figure 12. In the first instance, the shoreline is characterised by small gradient values, which made it barely distinguishable in the image. In the second instance, a part of the vehicle constitutes the most distinct line in the image. Consequently, it is qualified as the shoreline.

**Figure 13:**
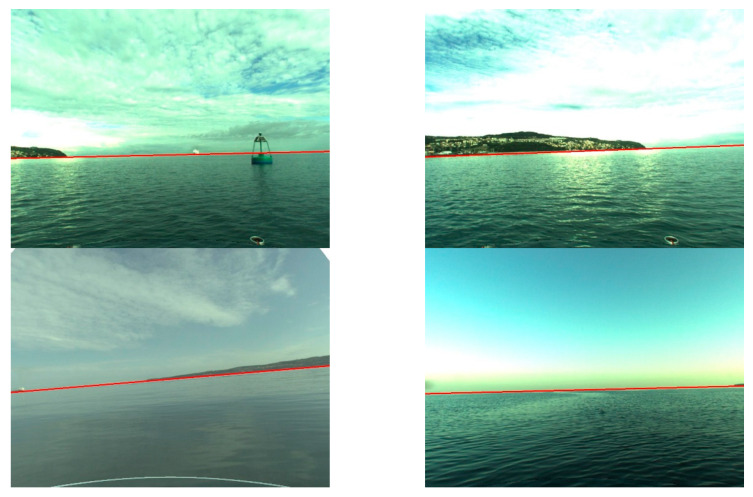
Results of shoreline detection using the Marine Obstacle Detection Dataset (MODD) database.

To determine accuracy, we decided to test our algorithm using the MODD database. This approach allowed us to compare the obtained results with other methods: Wang’s algorithm [10], MSCM-LiFe [21], and CFM [8]. Figure 13 presents some results obtained during the experiment.

The comparison procedure was adopted from [8]; consequently, mean height deviation (MHD) and mean angle deviation (MAD) were taken into consideration. Table 2 demonstrates that the presented method is more accurate than Wang’s algorithm and MSCM-LiFe in view of mean height deviation and gives better results than Wang’s algorithm considering MHD. Even though the presented method is worse than the CFS (coarse-fine-stiched), the obtained results indicate only small differences in obtained indicators.

**Figure 14:**
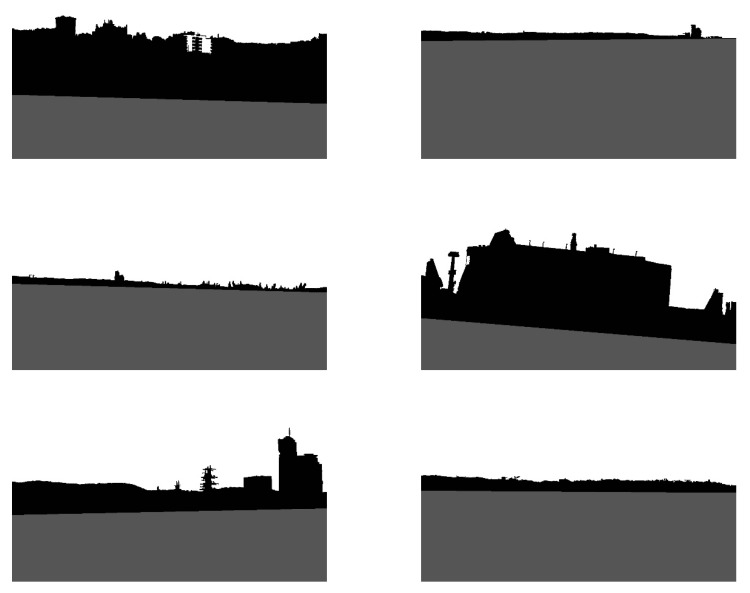
Results of land segmentation.

Secondly, land segmentation was performed. Exemplary outcomes are presented in Figure 14. 

In this case, the evaluation of obtained results was more complicated, since segmented images differed from desirable ones to varying degrees. For example, in Figure 14, it can be noticed that high-rise blocks are excluded from the land area. This is because the probabilistic clustering model qualifies them into the sky. However, the majority of the land is appropriately segmented. Therefore, to assess the obtained results, we have introduced a quality scale: segmented; partially segmented; not segmented. Segmented is when the entire land was correctly segmented. Partially segmented is when a minor part of the land was incorrectly segmented. Not segmented is when a significant part of the land was incorrectly segmented. The results, obtained by a human eye examination, are presented in Table 3.

It is apparent that in the majority of cases, the images are correctly segmented. Partial segmentation is mostly associated with including small parts of clouds to a land area or buildings and other infrastructure to sky. However, it slightly influences the obtained results because it involves minor areas of the segmented land. The improper segmentation appears when a shoreline is wrongly detected (see Figure 12). In these cases, only the sky area is defined correctly.

**Figure 15:**
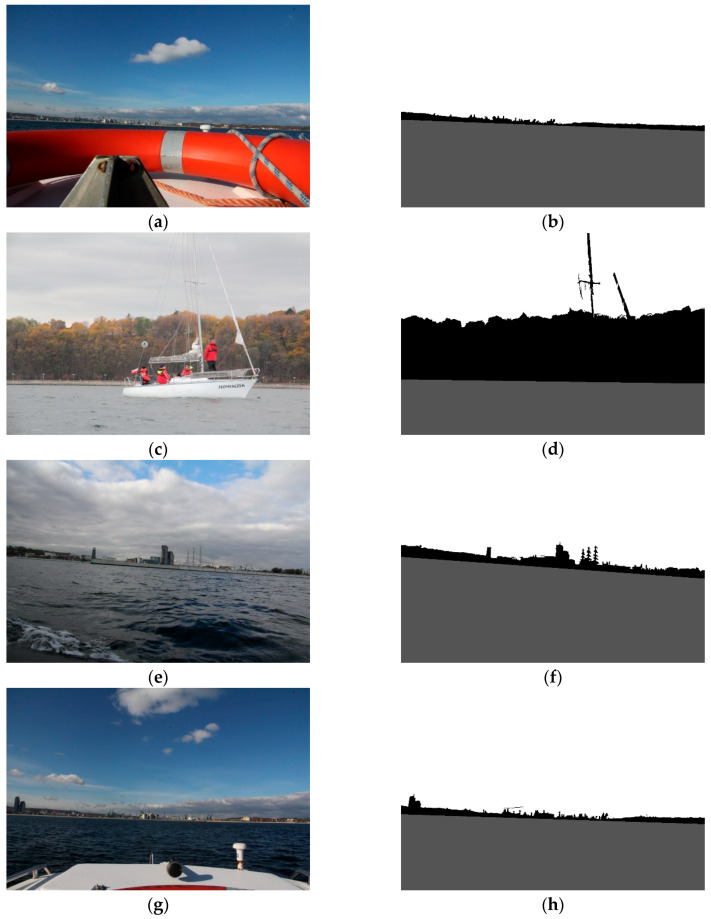
Results of land segmentation (**a**,**b**) The object presented in the sea area; (**c**,**d**) The object presented in the land area; (**e**,**f**) The shoreline does not expand to the whole image; (**g**,**h**) Partial segmentation due to the clouds.

The developed method performs correctly even if an object is visible in the sea area (Figure 15a,b), except when the object is defined by a straight line which could lead, erroneously, to merging it with the shoreline. Otherwise, any obstacle presented in land, sea and sky area, is included in the land area (Figure 15c,d). In some cases, when a shoreline is shorter and does not expand to the whole image, the longest visible segment is detected and spread across the image (Figure 15e,f). In Figure 15g,h, a typical case of partial segmentation is presented. Since the clouds appear close to the land area, and they constitute a patchy region, some parts of them are included in land.

The proposed method was considered in comparison to other methods that are based on traditional image processing. A technique that was developed for land segmentation in marine images was presented in [17]. The gradual edge level decrease method (GELD) was tested using the same database as in our research. Consequently, the obtained results are comparable. Other selected methods represent fundamental approaches to image segmentation in a wide range of applications. The following techniques were taken into consideration: Flood Fill, Watershed, Grabcut, Mean Shift, and K-means. On the grounds of the preliminary research, we established that the Flood Fill, Watershed, Grabcut and K-means techniques could be applicable for land detection. The Mean Shift technique groups pixels regardless of their position in the image; consequently, it cannot be used to segment complex scenes, such as a land area. Additionally, we discovered that land segmentation is only feasible when the image is initially divided into marine and continental regions. Therefore, we utilised the shoreline detection step to distinguish the sea area. Additionally, we resigned from testing of K-means technique, since its reduced viability for land detection was previously determined in [17]. The experiments were carried out using the developed database.

**Figure 16:**
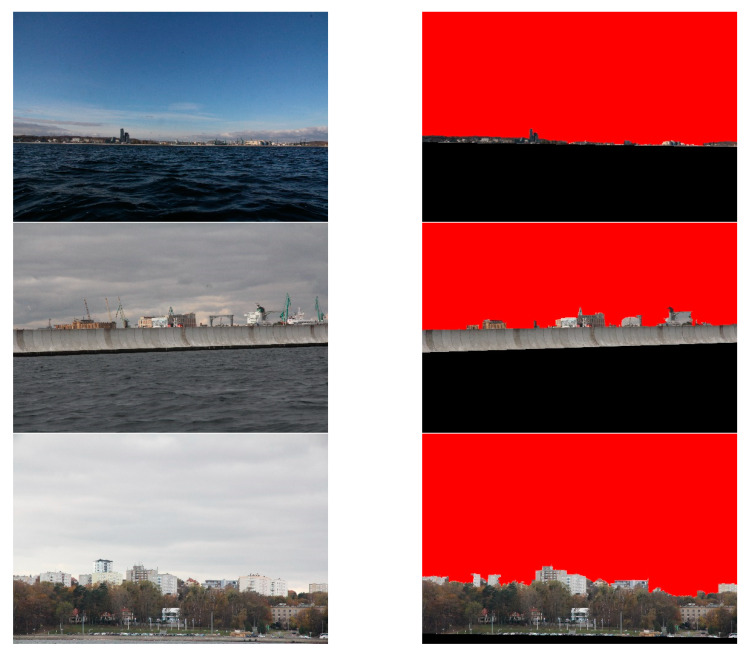
Results of the Flood Fill algorithm.

The Flood Fill method is often used to mark a portion of an image. This method constitutes selecting a seed point, to which all similar points are connected in the segment. In execution, four parameters are needed—first, minimal and maximal values of differences in pixel brightness. Then, two threshold parameters for the Canny detector. During the preliminary experiments, we found that the values 3, 3, 10 and 150, respectively, were appropriate for the majority of the images. In our investigation, we located the seed point in the upper left corner of the image. This was possible due to the assumption that a sky area is always visible on the picture. The results of the Flood Fill segmentation are presented in Figure 16.

The obtained results point out that the Flood Fill algorithm performs only when land is at a greater distance from the camera. However, it is prone to merge land and sky areas in case of shorter distances. Additionally, it incorrectly segments obstacles which are in front of a shoreline. The quantitative results, summarised in Table 4, indicate that the Flood Fill technique yields poorer image segmentation than the method under consideration.

**Figure 17:**
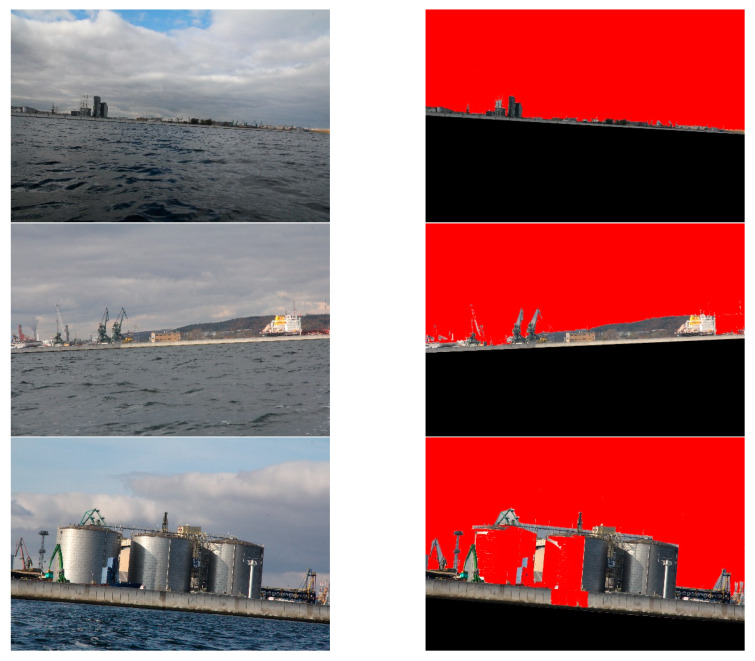
Results of the Watershed algorithm.

The Watershed algorithm converts edges in an image into “mountains” and plain regions into “valleys”. It successively floods basins starting from defined points until the areas meet. In this way, the basins connected to the marker point are segmented into the corresponding region. In our implementation, we establish that marker points, defining the sky area, lie on the line located 10 pixels below the upper edge of the image. The markers defining the land area constitute line, placed 10 pixels above the shoreline. By assuming that, the Watershed algorithm could be applied to determine the sky and land area. Figure 17 illustrates the obtained results.

The Watershed method is more prone to include land into the sky area than the Flood Fill algorithm and the method under consideration. This is the product of grouping strong edges, which divide images inside the land area. Consequently, in many cases, the images are incorrectly segmented. Statistically, the obtained results are summarised in Table 5.

**Figure 18:**
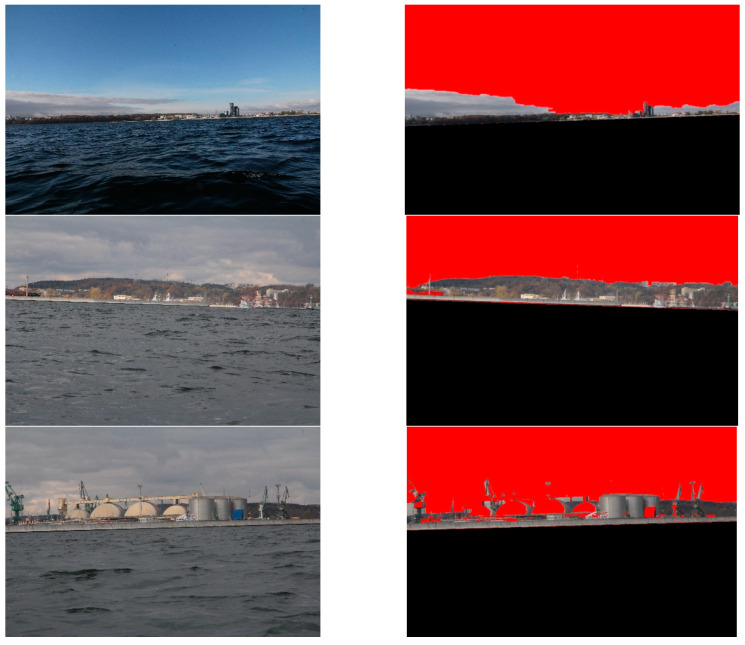
Results of the Grabcut algorithm.

The Grabcut algorithm was introduced by Rother, Kolmonogov, and Blake [30] as an extension of the Graphcut technique for use in user-directed image segmentation. In essence, the Graphcuts algorithm applies user-labelled foreground and background regions to establish distribution histograms. Therefore, since the unlabelled foreground and background should conform to the similarity distribution, an energy function that gives low energy for smooth and connected regions is in effect. What the Grabcut algorithm does is replace the histogram model of the Graphcut with Gaussian mixture one. Additionally, it solves the minimalisation problem iteratively and allows greater flexibility in the labelling process. In our application, we use the same labelling technique as in the Watershed method. The exemplary results are displayed in Figure 18.

They show that the Grabcut algorithm is sensitive to strong edges in the sky area. Additionally, it is prone to dividing the land area in case of a complex scene. The quantitative statistics, summarised in Table 6, indicate that the Grabcut algorithm performs correct segmentation only for a small number of the tested images. This can result from the presence of edges in land and sky areas leading to an erroneous partitioning of local regions.

The comparison of the tested algorithms is provided in Table 7. The segmentation quality was measured using the ratio of segmented images to whole images in the database. 

The performed analysis indicates that the proposed method precedes other methods based on traditional image processing technique. This derives from the fact that progressive land segmentation facilitates better separation between land and sky areas. In the first step of the presented method, all edges are taken into consideration to distinguish a land region. Then, weaker edges are removed, and the probability of an emerging area belonging to land is calculated utilising a probabilistic clustering model. In contrast, other methods assume that the most distinct lines always separate land and sky. However, in some cases, these lines are located inside land or sky regions. Only the GELD method considers the geometrical position of the sky–land line assuming the line extends through the entire image. However, it should be noted that in the case of the Flood Fill, Grabcut and Watershed techniques, only basic implementation was taken into consideration. This was due to the fact that any modification of these algorithms for land segmentation was not present in the literature. It is worth mentioning that the comparison with neural network methods was intentionally left out because its implementation process demands a large base of labelled images. Alternatively, we have noticed that the presented approach has built-in labelling images capability. Even though some images were only partially segmented, the outcome of progressive segmentation constitutes *T* images with changes in image depiction between one image and the other. Consequently, a human can decide which one constitutes the best segmentation. As the labelling task can be greatly simplified due to this segmentation, future work will be devoted to developing a database of labelled images and performing comparative analyses of traditional and neural network approaches.

## 4. Conclusions

Prior work has documented the importance of shoreline detection and land segmentation for ASV applications. Both solutions, based on traditional image processing and neural network, have been implemented for this purpose. On the one hand, traditional methods very often demand a set of parameters which should be established before execution. On the other hand, neural networks require an extensive database of labelled images. Therefore, this study developed a solution that, based on adaptive filtering and progressive segmentation, eliminates the need to set many parameters prior to execution.

An experimental investigation of the proposed method utilised one thousand five hundred (1500) images of Gdynia city, acquired in a potential region of a vehicle’s operation. The images show coastal areas at different distances from land and under various weather conditions. We found that, in the majority of cases, the developed method correctly performs shoreline detection and land segmentation. The comparison with other traditional image processing algorithms, such as the GELD, Flood Fill, Grabcut and Watershed algorithms, indicates that it features higher reliability. To improve the performance of the presented method in the progressive segmentation step, an investigation into the implementation of neural networks rather than the probabilistic clustering model will be conducted.

A neural network method was not compared to the method developed in this study. This was due to its need to possess an extensive database of the labelled images. However, since the outcome of progressive segmentation constitutes *T* images with different segmentation results, they can be used to simplify the labelling task. In this case, a human can decide which image represents the best segmentation. For that reason, future work will focus on developing shoreline detection and land segmentation methods based on neural networks as well as comparing them with the presented one. 

## Figures and Tables

**Table 1:** Results of shoreline detection.

Distance	Correctly Detected	Incorrectly Detected
Low	336	0
Medium	548	0
High	614	2

**Table 2:** Accuracy of the compared methods.

Method	MHD	MAD
Proposed Method	1.05	0.24°
Wang’s Algorithm	1.79	0.38°
CFS	0.89	0.19°
MSCM-LiFe	1.08	0.23°

**Table 3:** Results of land segmentation.

Distance	Segmented	Partially Segmented	Not Segmented
Low	324	12	0
Medium	523	25	0
High	585	29	2

**Table 4:** Results of land segmentation using the Flood Fill method.

Distance	Segmented	Partially Segmented	Not Segmented
Low	36	35	265
Medium	26	23	499
High	475	77	64

**Table 5:** Results of land segmentation using the Watershed algorithm.

Distance	Segmented	Partially Segmented	Not Segmented
Low	11	7	318
Medium	35	11	502
High	82	37	497

**Table 6:** Results of land segmentation using the Grabcut algorithm.

Distance	Segmented	Partially Segmented	Not Segmented
Low	6	7	323
Medium	20	16	512
High	51	48	517

**Table 7:** The precision of compared methods.

Distance	Proposed Method	GELD	Flood Fill	Watershed	Grabcuts
	[%]	[%]	[%]	[%]	[%]
Low	96	52	11	3	2
Medium	95	40	5	6	4
High	95	8	12	13	8
